# Supplementary material for: Elevated surface La promotes hyperfusion and contributes to impaired resorption in osteopetrosis
Source: bioRxiv. 2025 Sep 8:2025.09.07.674639. Preprint. [Version 2] doi: 10.1101/2025.09.07.674639 (PMC12440038; doi:10.1101/2025.09.07.674639)
Supplement: Supplement 1 [file NIHPP2025.09.07.674639v2-supplement-1.pdf]

bioRxiv preprint doi: https://doi.org/10.1101/2025.09.07.674639; this version posted September 8, 2025. The copyright holder for this preprint (which was not certified by peer review) is the author/funder. This article is a US Government work. It is not subject to copyright under 17 USC 105 and is also made available for use under a CC0 license.

SUPPLEMENTARY DATA

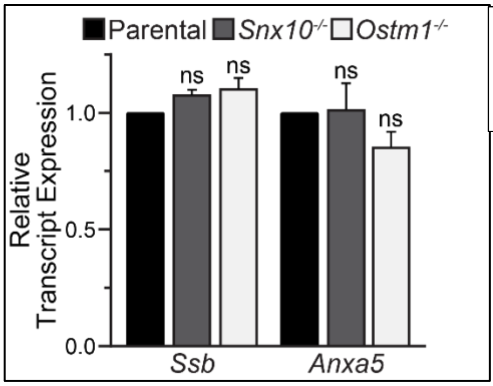

Supplementary Figure 1. Quantification of the steady-state transcript levels of the osteoclast fusion regulators *Ssb* and *Anxa5* relative to *GAPDH*. n = 5. p = 0.06, 0.08, 0.95, and 0.21, respectively.
